# Seroepidemiology of Human Bocaviruses 1 and 2 in China

**DOI:** 10.1371/journal.pone.0122751

**Published:** 2015-04-29

**Authors:** Yexia Hao, Jimin Gao, Xiaohong Zhang, Na Liu, Jinsong Li, Lishu Zheng, Zhaojun Duan

**Affiliations:** 1 Key Laboratory for Medical Virology, Ministry of Health, National Institute for Viral Disease Control and Prevention, China CDC, Beijing, China; 2 Wenzhou Medical University, Zhejiang, China; 3 National Institute of Sport Medicine, Beijing, China; Kliniken der Stadt Köln gGmbH, GERMANY

## Abstract

Seroepidemiology studies had been used to research the newly discovered human bocaviruses (HBoVs). Antibodies against the HBoV1–4 VP2 protein virus-like particles (VLPs) were found to be cross-reactive. The aim of the present study was to characterize the seroprevalence of HBoV1 and 2 among healthy populations in China. Recombinant HBoV1 and 2 VLPs were used to establish enzyme-linked immunosorbent assays (ELISAs) for detection of cross-reactivity between HBoV1 and HBoV2 in 1391 serum samples collected from healthy individuals in China. Of these, 884 samples were collected from Beijing and 507 were from Nanjing. Infection with HBoV1 and 2 was prevalent in healthy Chinese people, with the seroprevalence of HBoV1 and 2 in Beijing at 69.2 (612/884) and 64.4% (569/884), respectively. Highest seroprevalence was observed in 3–5-year-olds. The seroprevalence of HBoV1 was significantly decreased between 10–13-year-olds (80.3%) and 14–20-year-olds (62.3%, *p*< 0.05). For individuals over 20 years, seroprevalence was relatively constant at about 60%. Similar trends were observed in children from Nanjing, with seroprevalence of HBoV1 and 2 for healthy children at 80.7% (409/507) and 81.3% (412/507), respectively. Moreover, both mouse and human antibodies against HBoV1 and HBoV2 VLPs were found to be cross-reactive and 58.4% (813/1391) serum samples were seropositive for both HBoV1 and HBoV2. This finding suggests HBoV is highly prevalent in China and the antibodies produced as a result of infection with either HBoV1 or HBoV 2 will offer future protection. The cross-reactivity between HBoVs is crucial for accurately determining HBoV seroepidemiology.

## Introduction

Human bocavirus 1 (HBoV1) was discovered in 2005 in infants and children with infections of the lower respiratory tract [1]. Phylogenetic analysis showed the HBoV genome was most closely associated with canine minute virus (CMV) and bovine parvovirus (BPV). During 2009–2010, three other HBoV were discovered in stool samples, and from phylogenetic analysis these were designated HBoV2–4 [2–4]. HBoV1 is associated with respiratory tract disease [1, 5, 6], however, the exact pathogenic roles of HBoV1–4 are unclear.

Because there is no well-established cell culture system or animal model for HBoVs, diagnosis of HBoV infection relies on the detection of viral DNA using polymerase chain reaction assays. Seroepidemiology studies of HBoVs have mainly focused on HBoV1, such as the Western blot, immune fluorescence assays and Enzyme-linked Immunosorbent Assays (ELISA) had been used to detect IgG and IgM antibodies in serum specimens [7–19]. HBoV1-specific IgG antibodies are frequently detected in children, with 91.8% of infants in the first months of life found to be seropositive. Additionally, 40.7–60.0% of children between the ages of 5 and 47 months, and 85% of individuals 48 months and older, were seropositive for HBoV1-specific IgG antibodies [10]. It has been found that HBoV1 seroprevalence is age-related, and a greater number of healthy children were seropositive (84/233) compared with children that had lower respiratory tract infections (50/161) [12]. Kantola showed that antibodies against HBoV1–4 VP2 protein virus-like particles (VLPs) were cross-reactive, possibly accounting for the high HBoV1 seroprevalence previously reported [17].

The majority of epidemiological studies have been conducted on children or hospitalized patients with respiratory infections and are not representative of virus prevalence among the general population for all age groups [10–12, 18, 19]. In the present study, we prepared VP2 VLPs of HBoV1 and 2 and developed an ELISA to detect IgG antibodies against HBoVs.

## Materials and Methods

### Codon optimization of the HBoV1 and HBoV2 VP2 genes

In order to increase the yield of HBoV1 and HBoV2 VLPs, the HBoV1 VP2 (GenBank accession No. NC007455) and HBoV2 VP2 (GenBank accession No. NC012042) genes were optimized based on the codon preference for Sf9 insect cells. Two restriction enzyme sites, *Xba*I and *Hin*dIII, were included at the 5′ and 3′ ends of the synthesized genes, respectively. The optimization process and synthesis was conducted by Invitrogen (Beijing, China) and the optimized sequences were designated Y-HBoV1-VP2 and Y-HBoV2-VP2 for HBoV1 VP2 and HBoV2 VP2, respectively.

### Baculovirus expression of VP2

The sequences of VP2 genes were confirmed by sequencing and then inserted into the baculovirus expression transfer vector pFastBac1 (Invitrogen, Beijing, China) after digestion with an appropriate restriction enzyme. We followed the manufacturer’s recommended express protocol within the Bac-to-Bac expression kit (Invitrogen, Beijing, China). The generated transfer plasmid, pFastBac1-VP2, was transformed into *Escherichia coli* DH10Bac and the resulting recombinant baculovirus DNA (bacmid-VP2) was used to transfect Sf9 cells. After 3 days, infected cells were enlarged and stopped growing, therefore the supernatant of each culture was collected as the P1 viral stock. The baculovirus stock was amplified until the titer was >1 × 10^8^ pfu/mL and stored at 4°C until required. The titers of viral stocks were determined using plaque assays. The VP2 protein was expressed in Sf9 cells infected with the P4 viral stock (2 × 10^8^ pfu/mL) at a multiplicity of infection (MOI) of 5.0.

### Production of HBoV1 and 2 VLPs and immunization of mice

We infected Sf9 cells with recombinant baculoviruses and harvested cells at 7 days post-infection (dpi). Cells and supernatant were separated by centrifugation (1,000 × *g*, 20 min) and the VLPs in the supernatant were recovered by precipitation in 2.5% (v/v) polyethylene glycol 8000 (PEG8000; Sigma) for 3 h at 4°C with gentle agitation [20]. Cell pellets were resuspended in 25 mM NaHCO_3_ solution at a concentration of 2 × 10^7^ cells/mL and left on ice for 30 min to lyse cells. Supernatants containing VLPs were concentrated by precipitation in 2.5% PEG8000 as described above. The PEG-precipitated material was collected by centrifugation (6,000 × *g*, 45 min), and pellets were resuspended in phosphate-buffered saline (PBS, pH 7.4). Recombinant VLPs were purified by cesium chloride (CsCl) cushion centrifugation. The prepared protein samples were loaded on three cushions comprising CsCl solutions with densities of 1.5, 1.35 and 1.25 g/mL in PBS and subjected to ultracentrifugation (110,000 × *g*, 8 h, 4°C; Hitachi, Japan). The purified VLPs were negatively stained and pseudoparticles were observed using transmission electron microscopy (TECNAI 12, FEI, Blackwood, NJ, USA) with an acceleration voltage of 80 kV. Purified HBoV1 and HBoV2 VLPs were intramuscularly administered to BALB/c mice (25 μg/each mouse). Serum samples were collected at 7 weeks post-immunization. The animal experiment was carried out in accordance with the Guidelines for Animal Experiments described and approved by the Institutional Animal Care and Use Committee (IACUC) of Chinese Center for Disease Control and Prevention.

### ELISA detection of antibodies against HBoVs

Purified VLPs were used to establish an ELISA to detect IgG antibodies against HBoV1 and HBoV2. Briefly, 96-well plates (Corning, Beijing, China) were coated with VLPs at a concentration of 2.5 μg/mL in coating buffer (100 mM NaHCO_3_, pH 9.6) at 4°C overnight. Plates were washed three times with PBS-T (0.05% Tween 20 in PBS) and blocked by adding 200 μL of 5% (w/v) non-fat milk in PBS-T at 37°C for 1 h. Diluted serum samples (1:200) were added in duplicate and incubated for 1 h at 37°C. Following six washes with PBS-T, a 1:10,000 dilution of goat anti-human IgG conjugated to horseradish peroxidase (HRP; Kangwei Biotech, Beijing, China) was added to each well for 30 min at 37°C. Plates were incubated with substrate solution (Tiangen Biotech, Beijing, China) at room temperature for 10 min. 2 M H_2_SO_4_ was added to terminate the reaction and the optical density at 450 nm (OD_450_) was measured.

### Analysis of cross-reactivity using HBoV VLPs

The cross-reactivity between HBoV1 and HBoV2 was tested by ELISA. We coated 96-well plates (Corning, Beijing, China) with 100 μL of VLPs (2.5 μg/mL) overnight at room temperature. Plates were blocked by adding 200 μL of 5% (w/v) non-fat milk in PBS-T at 37°C for 1 h. HBoV1 antisera was added to 96-well plates coated with HBoV2 VLPs while HBoV2 antisera were added to 96-well plates coated with HBoV1 VLPs. To detect HBoV1-specific antibodies, human and mouse antisera were diluted in PBS containing soluble unbiotinylated HBoV2 VLPs (30 μg/mL) and pre-incubated for 1.5 h at 4°C, then transferred into wells containing immobilized HBoV1 VLP, or vice versa [17]. Sera from individuals that were HBoV-negative and pre-immune mice served as negative controls and the OD_450_ was determined.

### Clinical samples

1391 serum samples from individuals in two different geographical areas were examined. We collected 884 samples in 2011 from 287 children at the Beijing CDC and 597 adults that visited the National Institute of Sport Medicine (Beijing, China). The ratio of males to females was 396:488. We collected a further 507 serum samples in 2011 from healthy children aged 0–14 years at the Nanjing Children’s Hospital, Jiangsu Province, China. All samples were collected after obtaining written informed consent from parents or guardians, and stored at -80°C. Our study was approved by the Ethical Committee of the Capital Institute of Pediatrics.

### Statistical analysis

Seroprevalence was evaluated using the χ2 test and a *p*-value less than or equal to 0.05 was considered significant.

## Results

### Production of HBoV1 and HBoV2 VLPs

Analysis of the recombinant bacmind-VP2 by PCR and restriction analysis confirmed that the VP2 gene was inserted in the bacmind and in the correct orientation. The VP2 capsid protein of HBoV1 and HBoV2 was expressed in Sf9 cells and assembled into particles. Using CsCl cushion centrifugation, an obvious band appeared in the gradient at a density of around 1.32 g/cm^3^ [12]. The results of our codon optimization suggest that the yield of the VLPs for HBoV1 and HBoV2 was improved significantly from 0.031mg/ml to 1.5mg/ml and from 0.028mg/ml to 1.3mg/ml. In addition, the purified numerous VLPs can be observed clearly by transmission electron microscopy and exhibited the appearance of parvoviruses with a diameter of approximately 25 nm (data not shown).

### ELISA detection of antibodies against HBoV1 and HBoV2

To define a cut-off value we used western blots to identify 30 serum samples that were negative for IgG antibodies against HBoV1 and 2. The mean OD_450_ value for negative serum samples plus three times the standard deviation was considered the cut-off value. The ELISA cut-off value was 0.31 for HBoV1 and 0.3 for HBoV2.

### Seroprevalence of HBoV1 and HBoV2

Antibodies against HBoV1 and HBoV2 were detected in 1391 serum samples from healthy individuals. The seroprevalence of HBoV1 and 2 in Beijing (healthy individuals aged 0–84 years) was 69.2% (612/884) and 64.4% (569/884), in Nanjing (healthy children aged 0–13 years) was 80.7% (409/507) and 81.3% (412/507), respectively. Furthermore, 58.4% (813/1391) serum samples were seropositive for both anti-HBoV1 IgG and anti-HBoV2 IgG.

Among the 884 samples from Beijing, we found that 80% (88/110) of samples from infants (0–1 years) were seropositive for anti-HBoV1 IgG. The highest level of seroprevalence was 100% (32/32) for individuals aged 3–5 years. The seroprevalence in individuals aged 1–2 and 6–9 years was 93.8 and 94.8%, respectively. Seroprevalence was significantly decreased between the groups aged 10–13 years (80.3%) and 14–20 years (62.3%, *p* < 0.05). For individuals over 20 years, seroprevalence was relatively constant (about 60%) and then increased to 71.4% (95/133) in individuals older than 60 years.

**Fig 1:**
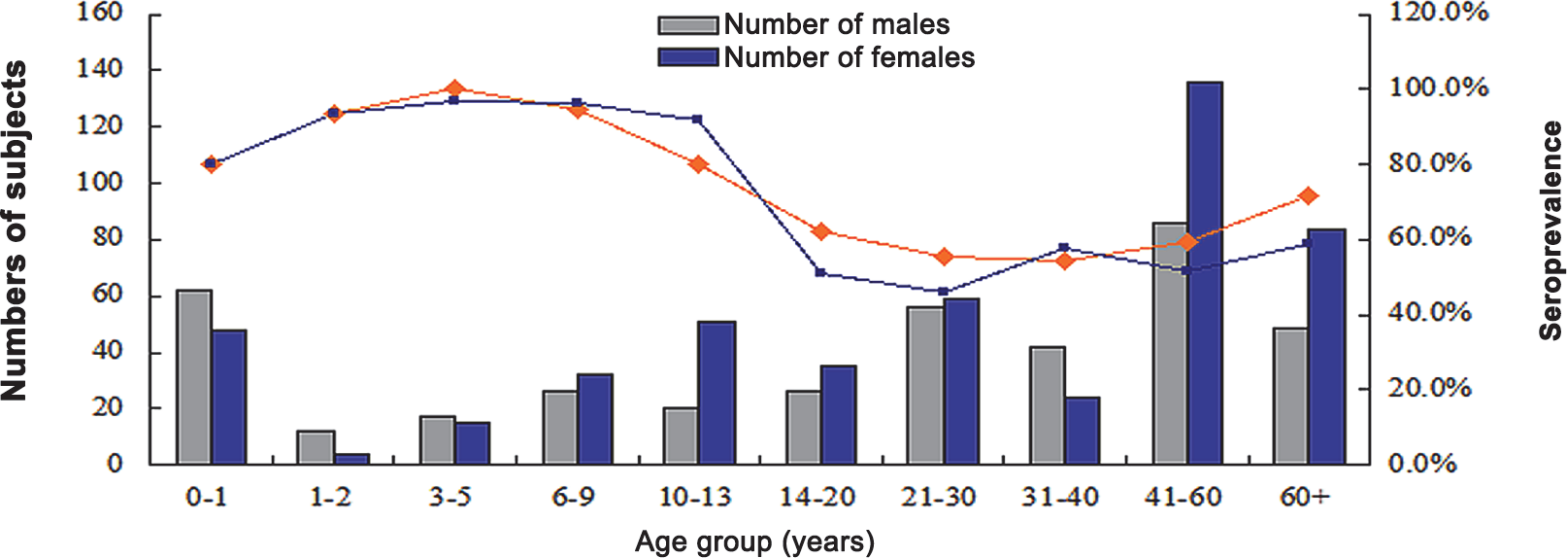
Seroprevalence of HBoV1 and HBoV2 by age group in Beijing.

Furthermore, seroprevalence of HBoV2 was greatest in 3–5-year-olds at 96.9% (31/32). Seroprevalence in adults was lower than that in children: 50.8% (31/61) for 14–20-year-olds; 46.1 (53/115) for 21–30-year-olds; 57.6% (38/66) for 31–40-year-olds; and 58.6% (78/133) for those older than 60 years (Fig 1). Although HBoV2 seroprevalence was higher than that for HBoV1 (88.9% *vs*. 86.1%) in 0–13-year-olds, this difference was not significant (χ^2^ = 1.016, *p* = 0.313).

**Fig 2:**
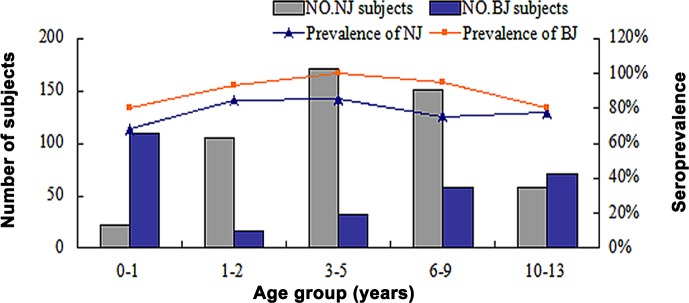
HBoV1 seroprevalence in children from Beijing and Nanjing.

Seroprevalence among the 507 healthy children from Nanjing revealed similar trends to those from Beijing (Fig 2). For infants (0–1 years), 68.2% (15/22) were positive for anti-HBoV1 IgG, increasing to 85.4% (146/171) in 3–5-year-olds and then decreasing to 77.6% (45/58) in 10–13-year-olds. HBoV2 seroprevalence decreased from 86.4% (19/22) in the 0–1-year-olds to 72.4% (42/58) in 10–13-year-olds (χ^2^ = 1.714, *p* = 0.190).

For all 1391 samples from Beijing and Nanjing, similar trends were observed between samples from males and females (χ^2^ = 1.28, *p* = 0.258). HBoV1 seroprevalence in children from Beijing and Nanjing was consistent (χ^2^ = 3.303, *p* = 0.069).

### Cross-reactivity of HBoVs

**Fig 3:**
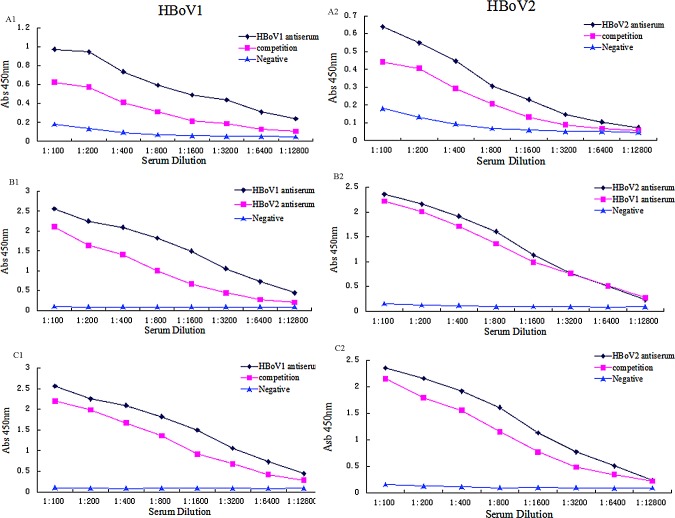
HBoV1 and 2 antisera showed cross-reactivity with HBoV1 and 2 VLPs. Negative human sera and pre-immune mice sera were used as negative controls. A1 and A2: human samples. B1, B2, C1, and C2: mouse samples.

Sequence alignment showed that the amino acid identity of VP2 was 77% between HBoV1 and 2 (data not shown). Antisera derived from humans and mice were used to analyze cross-reactivity between HBoV1 and 2 VLPs. Positive human antisera were identified by ELISA to contain antibodies against either HBoV1 or 2 but not both. HBoV1-positive antisera reacted with HBoV2 VLPs, while HBoV2-positive antisera reacted with HBoV1 VLPs (Fig 3). The OD_450_ for HBoV1-reactive antibodies decreased after depletion with HBoV2 VLPs and vice versa.

## Discussion

Currently, diagnosis of HBoV infections mainly relies on PCR assays with various genes (NP1 [1, 21, 22], NS1 [22–25], and VP1/VP2 [26–28]) targeted. Seroepidemiology studies have been performed to study the primary features of HBoVs. However it is not possible to propagate HBoVs in cell culture or in experimental animals, therefore VLPs are an ideal antigen for seroepidemiological investigations. In our study, VLP expression was increased by optimizing codons in the VP2 genes of HBoV1 and 2. Compared with the production without codon optimization, the yield of the VLPs for HBoV1 and HBoV2 was improved markedly after codon optimization. Meanwhile, the purified VLPs can be observed using transmission electron microscopy much more numerously and clearly.

Seroprevalence in the various age groups exhibited similar trends to those seen previously [9–11, 29]. The overall seroprevalence of HBoV1 (69.2%) observed in Beijing was consistent with that seen in previous serological studies conducted in Japan (71.1%) [9] and Jamaica (76.7%) [16], but higher than that previously reported for Beijing (59.1%) [29]. This is possibly due to differences in the age group structure for the various studies. Prevalence of HBoVs IgGs was high in healthy children, with HBoV1 seroprevalence in healthy children 12 months and younger 80.0% (Beijing) and 68.2% (Nanjing), indicating that HBoV1 infection is common during childhood and could be caused by the presence of maternal antibodies. HBoV infection in children clearly occurs frequently as virus-specific antibodies last longer than viruses in a patient after any other acute viral infection [12].

The 61.1% (365/597) seroprevalence of HBoV1 in adult individuals (≥15 years old) was lower than that previously reported [17, 30], especially in Beijing 99.3% [31], but consistent with that for the United States (63%) [20]. However, the prevalence of HBoV1 and 2 in 5–14-year-olds (86.8 and 93.8%, respectively) was higher than previously reported (77.9 and 61.1%, respectively) [31], possibly because of differences in sampling.

The seroprevalence of HBoV1 in healthy children from Nanjing was similar to that seen in Beijing, increasing from 68.2% (15/22) for 0–1-year-olds to 85.4% (146/171) for 3–5-year-olds. Results from previous studies showed that the proportion of HBoV1-seropositive individuals increased to greater than 85% in children older than 4 years [10]. It is likely some of these children were exposed to HBoV1 in day care or preschool environments, which parallels the primary period of parvovirus B19 infection [32, 33].

There are few seroprevalence studies regarding HBoV2. Current detection methods focus on the use of PCR assays to detect HBoV2 in respiratory tract and stool samples. In China, using real-time PCR assays, it was previously shown that 20.4% (129/632) of children with diarrhea and 12.3% (20/162) of healthy children were positive for HBoV2 [34]. In our study, the seroprevalence of HBoV2 in children from Beijing was 64.4% (569/884) and 81.3% (412/507) for Nanjing. This discrepancy is likely because the PCR results only reflect an ongoing infection, whereas ELISA results reflect the exposure rate of accumulated infections. Seroprevalence in our study was higher than that observed by Kantola *et al*., in which the prevalence specific anti-VP2 IgG antibodies against HBoV 1–4 was detected after depletion of HBoV1-reactive antibodies [17].

Because of the high amino acid identity for VP2 between HBoV1 and 2, cross-reactivity of antisera against HBoV1 and 2 VLPs has been reported.To eliminate the interference of antibody cross-reactivity, the antigen competition ELISA (cELISA) assay was established by Guo *et al*. Differential seroprevalence of HBoV 1–4 had been reported in China with/without an antigen cELISA. Without competition, more than 90% samples were positive for HBoV1, HBoV2, and HBoV3; 73 (51.4%) samples were positive for HBoV4. With the cELISA by VLPs of heterologous HBoV species, these IgG seroprevalences decreased dramatically to 66.9%, 49.3%, 38.7%, and 1.4%, for HBoV1, 2, 3, and 4, respectively [31].

In our study, the OD_450_ for HBoV1 antibodies decreased after pre-incubation with HBoV2 VLPs and vice versa. These findings further indicate a high degree of antigenic cross-reactivity between HBoV1 and HBoV2, therefore the seroprevalence of HBoV1 and HBoV2 in this report could be overestimated. The seroprevalence of HBoV1 and 2 in 1391 samples derived from Beijing and Nanjing was 73.4% (1021) and 70.5% (981) respectively. Particularly, the seroprevalence of HBoV1 among children aged from 1–9 years old was above 90%. The serological cross-reactivity of HBoV1 with HBoV2–4 partially accounts for the high HBoV1 seroprevalence reported. Additionally, these data suggest that infection with either HBoV1 or 2 will protect against HBoV3 or 4 infections.

Notably, Gorilla Bocavirus species 1 (GBoV1), the first distinct species of non-human primate bocavirus, was identified in stool samples of gorillas with acute enteritis from a captive colony, and showed a high sequence similarity to HBoV1 [35]. GBoV1 and its related variant were detected in stool samples of wild animals in captivity for several years, suggesting bocavirus infection may be persistent in those animals [36]. Whether HBoV DNA persists in tumors as episomes or whether it is integrated into the human genome has been investigated recent years. The head-to-tail DNA replication intermediates, chemically defined as a covalently closed circular DNA, of HBoV were observed in clinical samples and cell culture supernatant, and these findings led to the hypothesis that HBoV DNA replication may be different from other parvoviruses [37]. Subsequently, the head-to-tail sequences for HBoV1 was confirmed by Kapoor and colleagues, it was reported that HBoV3, like other well-known parvoviruses, can exist as episomes in infected human intestinal tissue and therefore can likely establish persistent infection in the host [38–40]. In addition, the HBoV DNA can also be detected in tumor tissue samples from lung and colorectal cancers and persist in solid tumors [41]. This finding suggested that HBoV, by analogy to the hepatitis B virus, may indirectly contribute to the development of some cancers or may play an active role in human cancers. The high percentage of viral DNA in samples from symptomatic patients and healthy subjects, and viral persistence in human tissue may explain the reason of high seroprevalence in different populations. We should pay more attention on the HBoV persistence, and cohort study of HBoV seroepidemiology is needed to determine the association of HBoV with disease.

In conclusion, the determination of HBoV1 and 2 seroepidemiology in healthy individuals as described in this study will assist with studying the prevalence of other newly discovered viruses in China. To obtain accurate data regarding the seroprevalence of all four HBoVs, it is necessary to consider the cross-reactivity of antisera against HBoVs.

## Supporting Information

S1 ARRIVE Checklist The ARRIVE Guidelines Checklist (Page 1). (JPG)

S2 ARRIVE Checklist The ARRIVE Guidelines Checklist (Page 2). (JPG)
