# Supplementary figures and images for: Seroepidemiology of Human Bocaviruses 1 and 2 in China
Source: PLoS One. 2015 Apr 29;10(4):e0122751. doi: 10.1371/journal.pone.0122751 (PMC4414540; doi:10.1371/journal.pone.0122751)

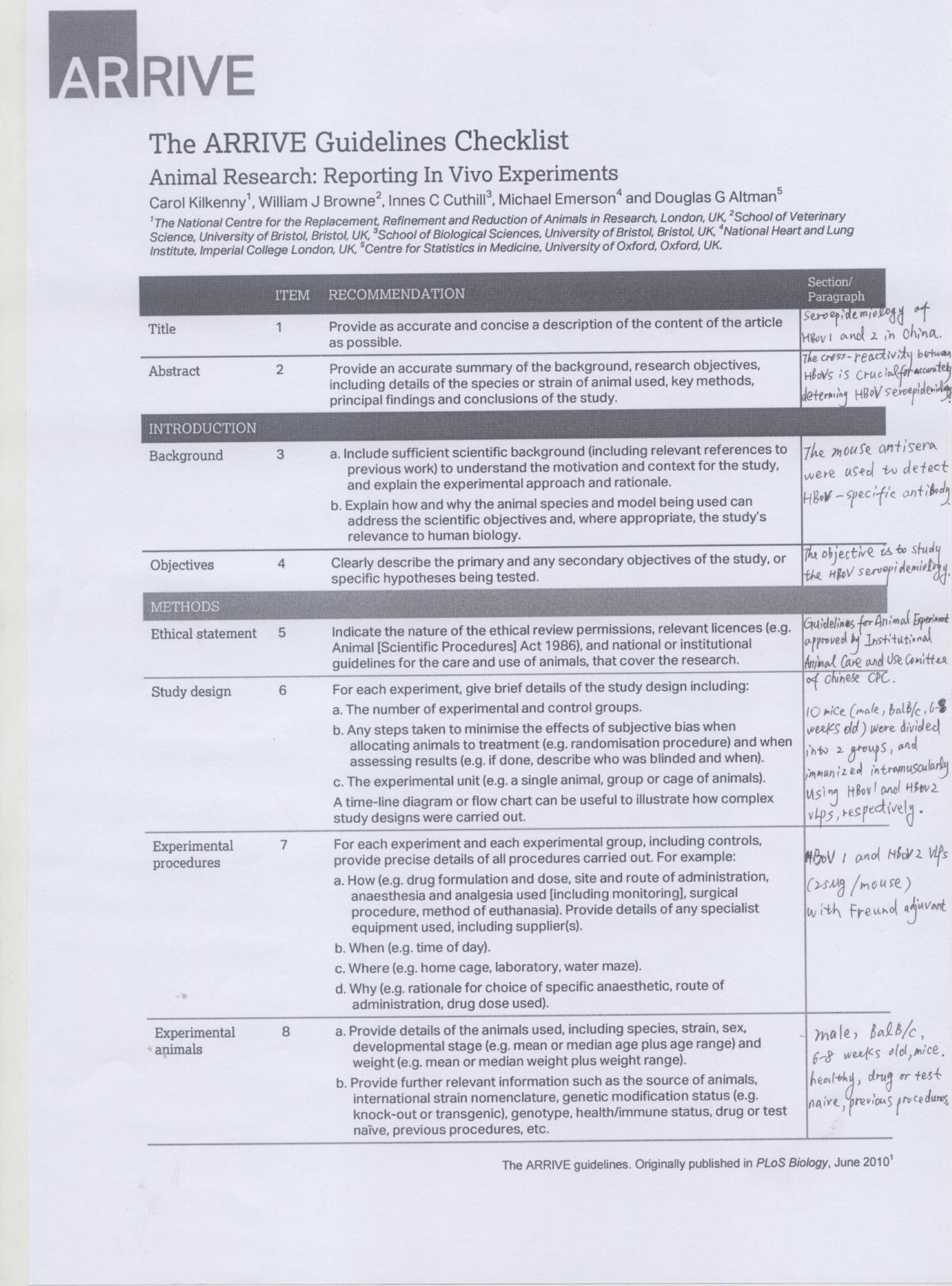

Supplement: S1 ARRIVE Checklist — (JPG) [file pone.0122751.s001.jpg]

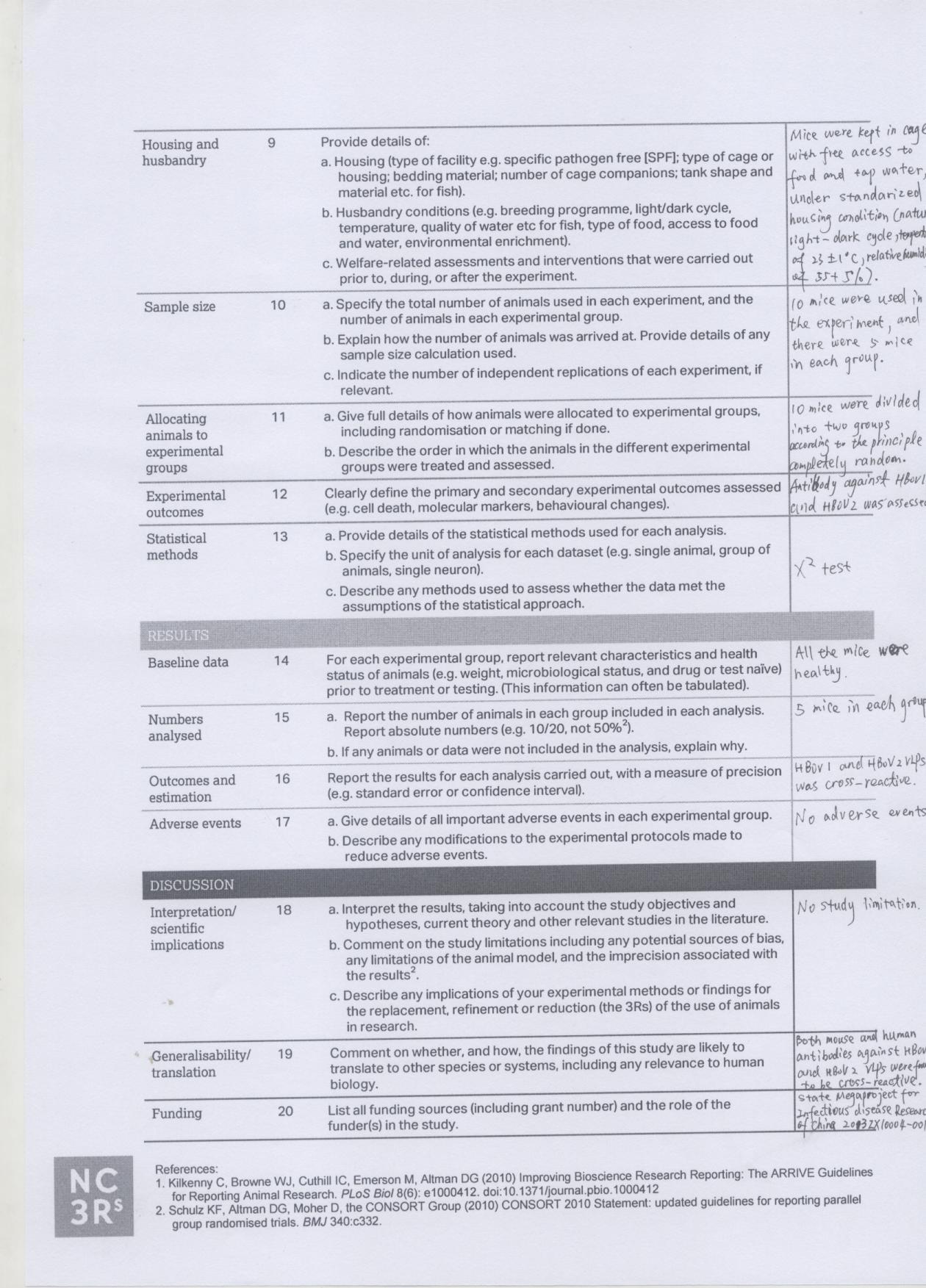

Supplement: S2 ARRIVE Checklist — (JPG) [file pone.0122751.s002.jpg]
